# Supplementary material for: GSTZ1 sensitizes hepatocellular carcinoma cells to sorafenib-induced ferroptosis via inhibition of NRF2/GPX4 axis
Source: Cell Death Dis. 2021 Apr 30;12(5):426. doi: 10.1038/s41419-021-03718-4 (PMC8087704; doi:10.1038/s41419-021-03718-4)
Supplement: Supplementary file 1 — Supplementary Materials [file 41419_2021_3718_MOESM1_ESM.docx]

**Supplementary Information**

**GSTZ1 sensitizes hepatocellular carcinoma cells to sorafenib-induced ferroptosis via inhibition of NRF2/GPX4 axis**

Wang et al

**^Contents^**

**Supplementary Figure 1.** GSTZ1 overexpression enhances sorafenib-induced ferroptosis in HCC.

**Supplementary Figure 2.** GSTZ1 S14A suppresses sorafenib-induced ferroptosis.

**Supplementary Figure 3.** GSTZ1 sensitizes hepatoma cells to sorafenib-induced ferroptosis through the NRF2 signaling pathway.

**Supplementary Figure 4.** RSL3 enhances the anticancer activity of sorafenib in *Gstz1*-knockout mice.

**Supplementary Table 1.** Primer sequences used in this study.

**Supplementary figures, table and legends**

**
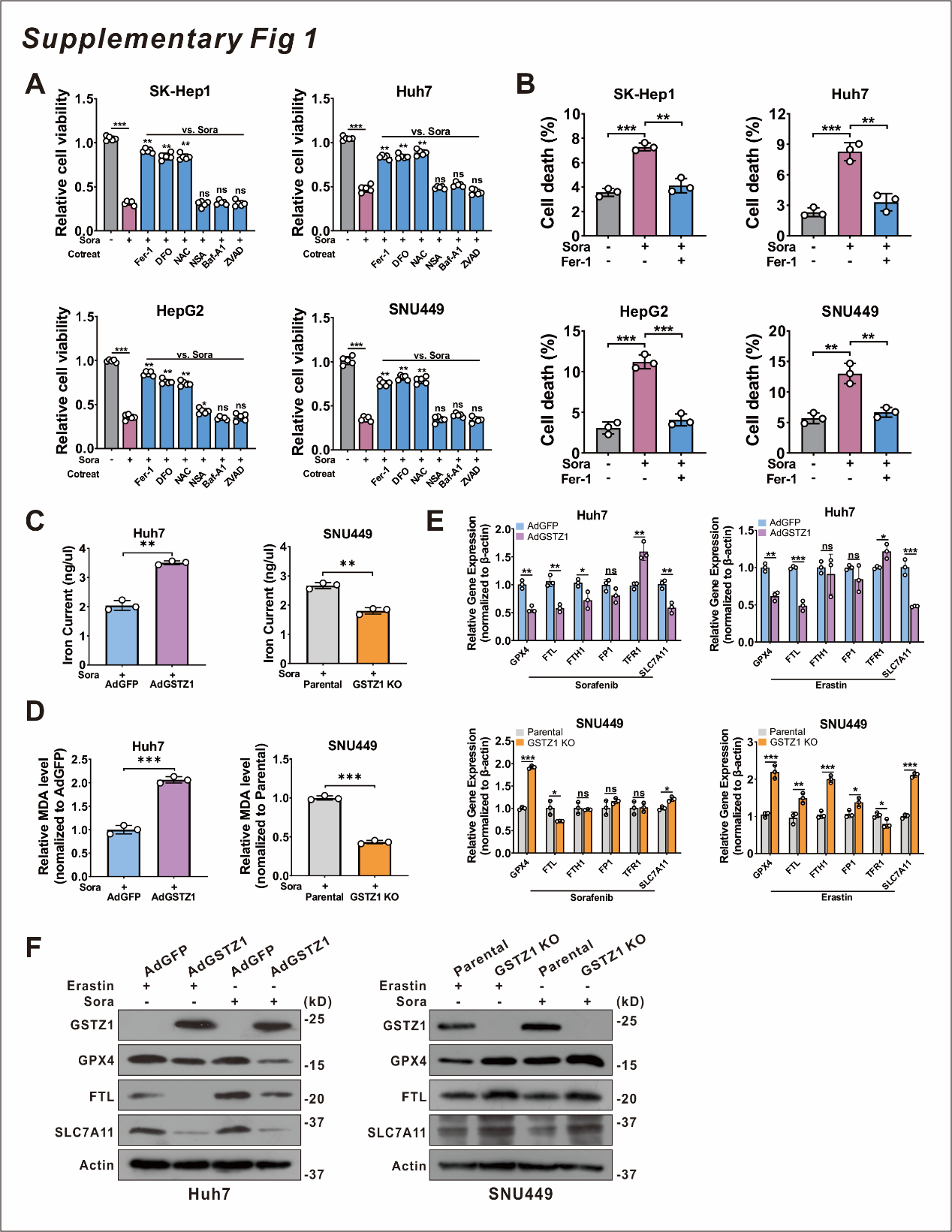
**

**Supplementary Figure 1.** GSTZ1 overexpression enhances sorafenib-induced ferroptosis in HCC. **a** Cell viability in HCC cells treated with sorafenib alone or in combination with Fer-1, DFO, NAC, NSA, Baf-A1, ZVAD (n = 5). **b** Cell death were analyzed by Annexin V/PI double staining and flow cytometry. **c-d** Intracellular iron (**c**) and MDA (**d**) levels in GSTZ1-OE and GSTZ1-KO cells treated with sorafenib for 24 h. **e-f** mRNA (**e**) and protein (**f**) levels of target genes associated with ferroptosis in GSTZ1-OE and GSTZ1-KO cells treated with sorafenib or erastin, determined via qRT-PCR and western blotting, respectively. For western blotting, 50 μg protein was loaded per well. HCC: hepatocellular carcinoma, Sora: sorafenib, MDA: malondialdehyde. Fer-1: ferrostatin-1, DFO: deferoxamine, NAC: N-acetyl-L-cysteine, Nec: Necrosulfonamide, Baf-A1: bafilomycin A1, ZVAD: ZVAD-FMK. Values represent the mean ± SD (n = 3). The qRT-PCR data determined from three independent experiments. ns: no significant difference, **p* < 0.05, ***p* < 0.01, ****p* < 0.001, Student’s t-test (two groups).


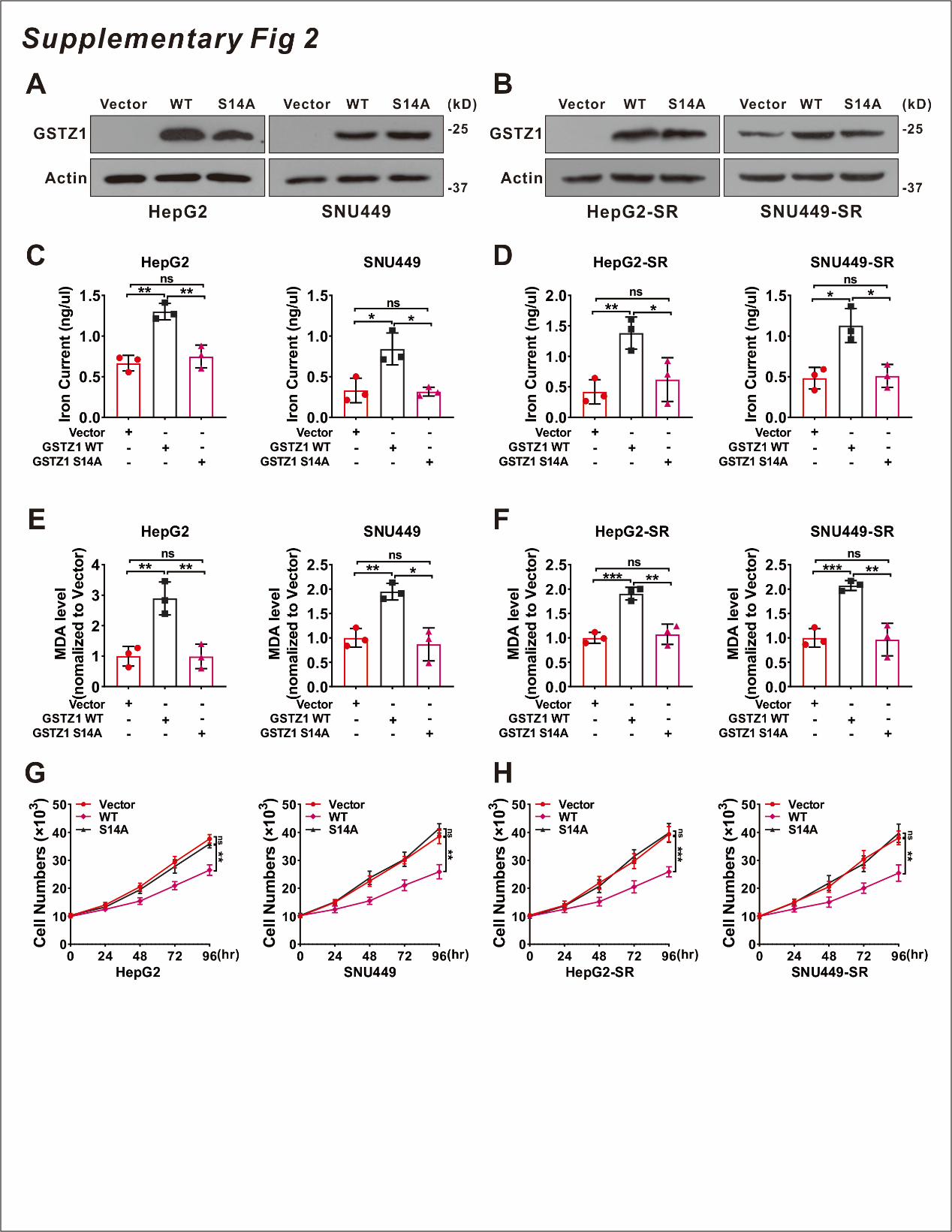


**Supplementary Figure 2.** GSTZ1 S14A suppresses sorafenib-induced ferroptosis. **a-b** GSTZ1 expression levels were analyzed by western blotting. **c-f** Iron (**c-d**) and MDA (**e-f**) levels in GSTZ1-KO and sorafenib resistant cells expressing wild-type (WT) or S14A mutant GSTZ1 upon sorafenib stimulation. **g-h**. Cell growth curve. For western blotting, 50 μg protein was loaded per well. WT: wild-type. SR: sorafenib resistant. Values represent the mean ± SD (n = 3). ns: no significant difference, **p* < 0.05, ***p* < 0.01, ****p* < 0.001, Student’s t-test (two groups).


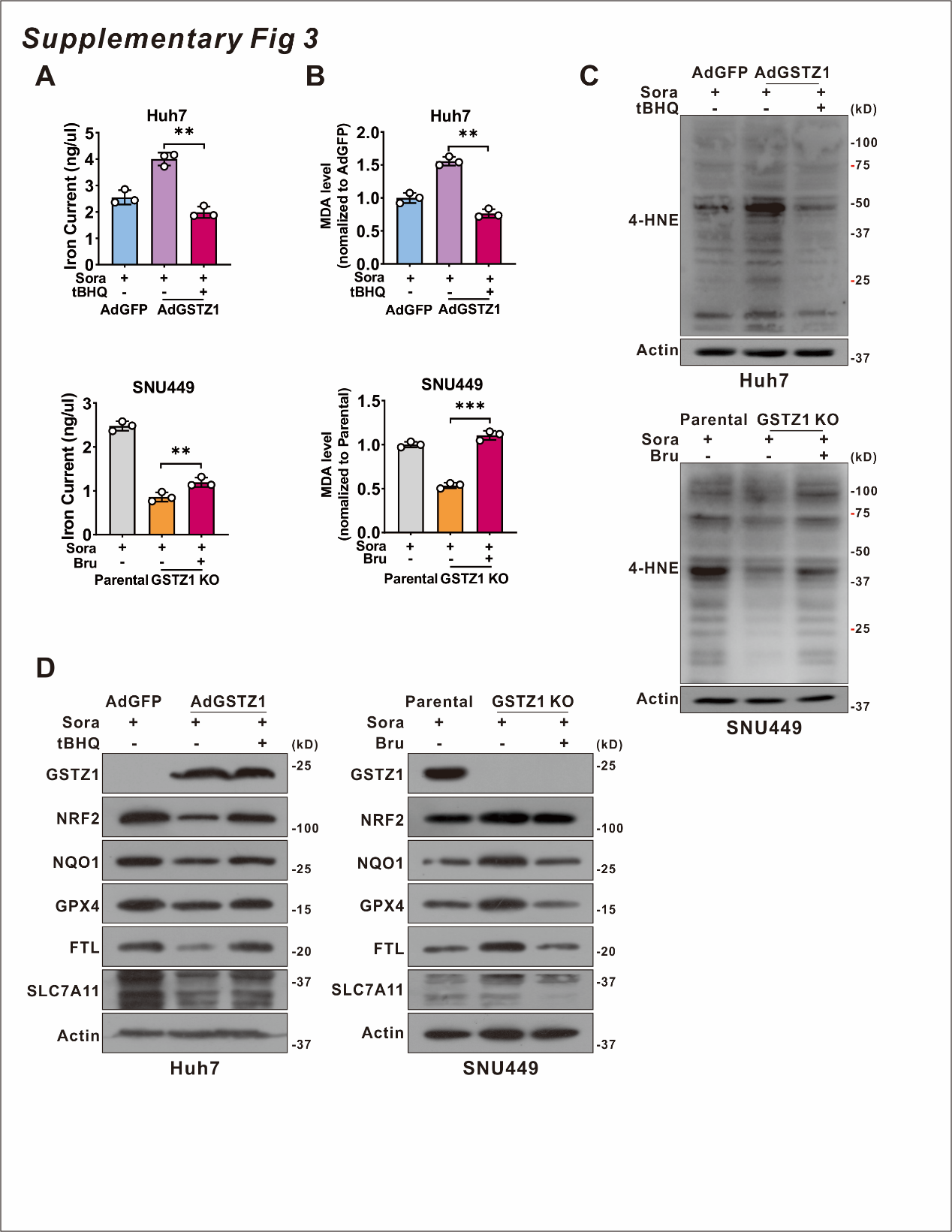


**Supplementary Figure 3.** GSTZ1 sensitizes hepatoma cells to sorafenib-induced ferroptosis through the NRF2 signaling pathway. **a-b** Levels of iron (**a**) and MDA (**b**) in GSTZ1-OE cells treated with sorafenib alone or in combination with tBHQ (top) and GSTZ1-KO cells treated with sorafenib alone or in combination with Bru (bottom). **c-d** Levels of 4-HNE modification and protein related with ferroptosis in GSTZ1-OE and -KO cells. The cell processing is described as above. For western blotting, 50 μg protein was loaded per well. tBHQ: tertiary butylhydroquinone, bru: brusatol, Sora: sorafenib, MDA: malondialdehyde, 4-HNE: 4-hydroxy-2-nonenal. Values represent the mean ± SD (n = 3). **p* < 0.05, ***p* < 0.01, ****p* < 0.001, Student’s t-test (two groups).


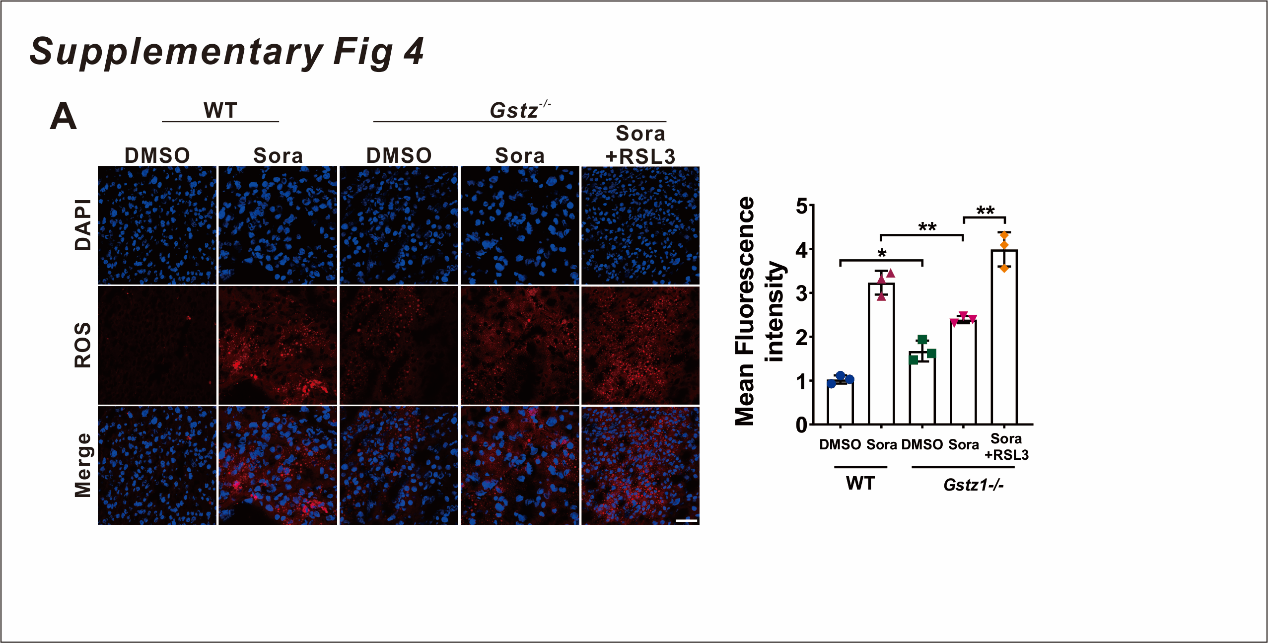


**Supplementary Figure 4.** RSL3 enhances the anticancer activity of sorafenib in Gstz1-knockout mice. **a** Representative fluorescence staining of ROS with CellROX Orange probe in hepatic tumors of five groups mice (left). Scale bar = 50 μm. Intracellular ROS quantification (right). Values represent the mean ± SD (n = 3), **p* < 0.05, ***p* < 0.01, one-way ANOVA followed by Tukey tests (five groups).

**Supplementary Table 1.** Primer sequences used in this study.

| Name | Accession Number | Source | Sequence (5’-3’) |
| --- | --- | --- | --- |
| *GSTZ1*  (Human) | NM_145870 | TsingKe Biological Technology | Forward: CCTGAAGCAAGTGGGAGAGG  Reverse: TGATGGTAGGGTAGGGGGTG |
| *GSTZ1 S14A*  (Human) | NM_145870 | TsingKe Biological Technology | Forward: TCCTATTTCCGAGCCTCCTGCTCATGGA  Reverse: TCCATGAGCAGGAGGCTCGGAAATAGGA |
| *FTL*  (Human) | NM_000146.4 | TsingKe Biological Technology | Forward: GATGATGTGGCTCTGGAAGGC  Reverse: TGTGGAGGTTGGTCAGGTGG |
| *GPX4*  (Human) | NM_002085.5 | TsingKe Biological Technology | Forward: CCGCCTTTGCCGCCTAC  Reverse: TTTACTTCGGTCTTGCCTCACT |
| *FTH1*  (Human) | NM_002032.3 | TsingKe Biological Technology | Forward: CGCCAGAACTACCACCAGG  Reverse: CAAAGAAGTCCTCCAGCTTG |
| *FP1*  (Human) | NM_014585.6 | TsingKe Biological Technology | Forward: TCATCGGCTGTGGCTTTATT  Reverse: CTGGGAGGCACAAGTAGGCT |
| *TFR1*  (Human) | NM_003234.4 | TsingKe Biological Technology | Forward: GCTTTCCCTTTCCTTGCATAT  Reverse: CACGAACTGACCAGCGACCT |
| *SLC7A11*  (Human) | NM_014331.4 | TsingKe Biological Technology | Forward: TTTCTGAGCGGCTACTGGG  Reverse: CAAAGGGTGCAAAACAATAACA |
| *β-actin*  (Human) | NM_001101 | TsingKe Biological Technology | Forward: AGGCCAACCGCGAGAAGATGACC  Reverse: GAAGTCCAGGGCGACGTAGCAC |
| *Gpx4*  (Mouse) | NM_008162.4 | TsingKe Biological Technology | Forward: GCAATGAGGCAAAACTGACG  Reverse: CCCTTGGGCTGGACTTTCA |
| *β-actin*  (Mouse) | NM_007393 | TsingKe Biological Technology | Forward: CGTTCAATACCCCAGCCATG  Reverse: GACCCCGTCACCAGAGTCC |
